# Novel therapeutic strategies for targeting fatty acid oxidation in cancer

**DOI:** 10.1186/s40364-025-00855-2

**Published:** 2025-11-11

**Authors:** Yan Wang, Mengsi Zhang, Jihong Liu, Chenglong Li, Na Sun, Xiujuan Wu, Chengfang Wang, Xuanni Tan, Yi Yang, Xiaowei Qi, Yi Zhang

**Affiliations:** 1 Department of Breast and Thyroid Surgery, Southwest Hospital, Army Medical University, Chongqing, 400038 P.R. China; 2 Key Laboratory of Chongqing Health Commission for Minimally Invasive and Precise Diagnosis and Treatment of Breast Cancer, Chongqing, 400038 P.R. China; 3 Institute of Pathology & Southwest Cancer Center, Southwest Hospital,and School of Basic Medical Sciences., Army Medical University, Chongqing, 400038 P.R. China; 4 Department of Respiratory and Critical Care Medicine, Southwest Hospital, Army Medical University, Chongqing, 400038 P.R. China

**Keywords:** Fatty acid oxidation, Cancer, Carnitine palmitoyltransferase 1, Targeted therapy, Tumor microenvironment

## Abstract

Metabolic rewiring is a defining feature of malignant cells, enabling them to dynamically exploit nutrient resources to meet bioenergetic problems at different growth stages. Beyond the classical Warburg effect, recent studies have shown that neoplasms demonstrate a marked dependency on lipid metabolism, using free fatty acids to support cellular proliferation and regeneration via fatty acid oxidation (FAO). As a central component of lipid metabolism, FAO exerts dual immunomodulatory functions within tumors. Although numerous studies have described the enzymatic reactions of the FAO pathway in different malignancies, relatively few have investigated the pharmacological disruption of these enzymatic checkpoints and the resulting immunological consequences. Moreover, existing therapeutic strategies have failed to achieve a risk–benefit balance, limiting the clinical translation of FAO-directed approaches. To better understand the therapeutic implications of FAO, we investigated the mechanistic pathways mediated by mitochondrial rate-limiting enzymes, with a particular focus on the carnitine palmitoyltransferase 1 enzyme family—the critical gatekeeper controlling the entry of fatty acids into mitochondrial oxidation instead of CPT2. We comprehensively evaluated its role in tumor biology and also highlight future research directions to inform rational intervention strategies.

## Introduction

**Fig. 1:**
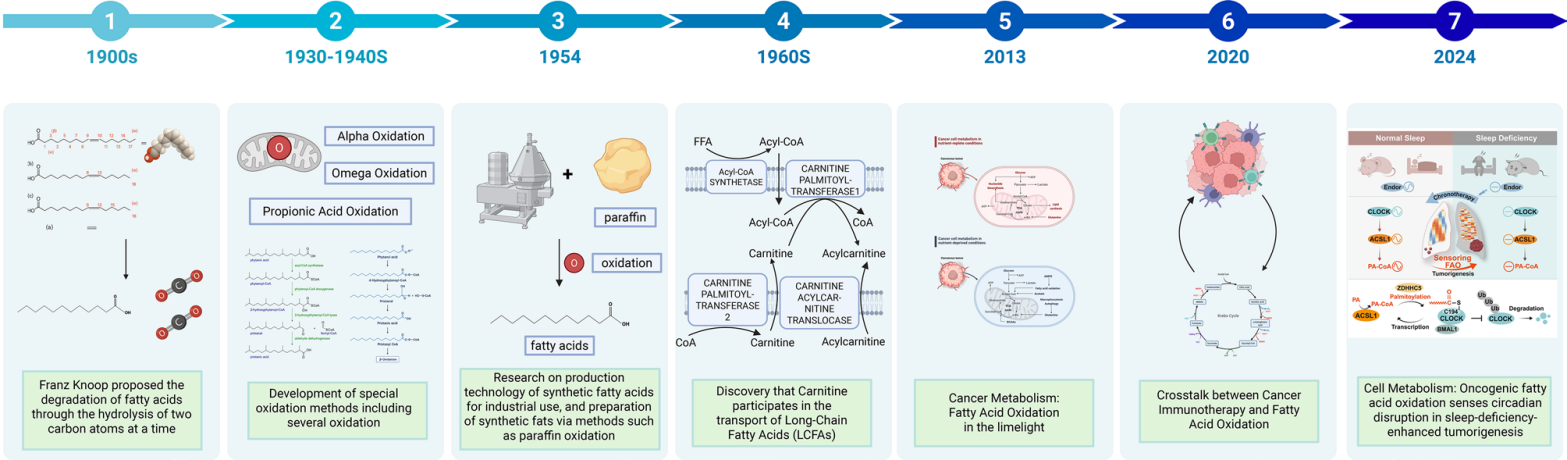
Timeline of FAO research. A systematic summary of the evolution of FAO, encompassing theoretical proposals, technical applications, and cancer-related studies over the past century

In the late nineteenth century, fatty acid oxidation (FAO) was recognized as a process of fatty acid degradation that happens in cycles of hydrolysis of two carbon atoms [1]. Later, other oxidation pathways were discovered, including pyruvate oxidation, α-oxidation, ω-oxidation [1, 2]. In the 1960s, carnitine was reported to play a key role in transporting long-chain fatty acids, thereby establishing β-oxidation as a central mechanism for maintaining membrane structure, supporting cell renewal, regulating hormones, and mediating signal transduction [3]. However, the relationship between FAO and cancer received little attention until much later [4]. Recent advances (2020–2024) have revealed that FAO participates in cancer immunotherapy and interacts with circadian rhythm regulation [5–7]. After more than a century of investigation, FAO research continues to expand, offering new opportunities to identify therapeutic vulnerabilities in cancer (Fig. 1).

FAO occurs in both peroxisomes and mitochondria. Peroxisomal FAO, regulated by acyl-CoA oxidase, is responsible for the degradation of long-chain fatty acids. In contrast, mitochondrial FAO, regulated by the rate-limiting enzyme carnitine palmitoyltransferase 1 (CPT1), generates large amounts of ATP for lipid metabolism. Hence, in this article, we focus on mitochondrial FAO, with particular emphasis on the CPT1 family.

Although CPT1-targeted therapies exhibit promising anticancer potential, their clinical development is limited by poor selectivity and severe adverse effects. Here we review the structural and functional features of CPT1, its physiological roles, regulatory mechanisms, and oncogenic implications, along with emerging strategies to overcome current barriers to drug development.

## CPT1 structure and function

CPT1 is the core enzyme in FAO [8, 9]. The mammalian CPT1 family comprises three homologs: CPT1A (liver-type), CPT1B (muscle-type), and CPT1C (brain-type) [10].

### Structure

**Fig. 2:**
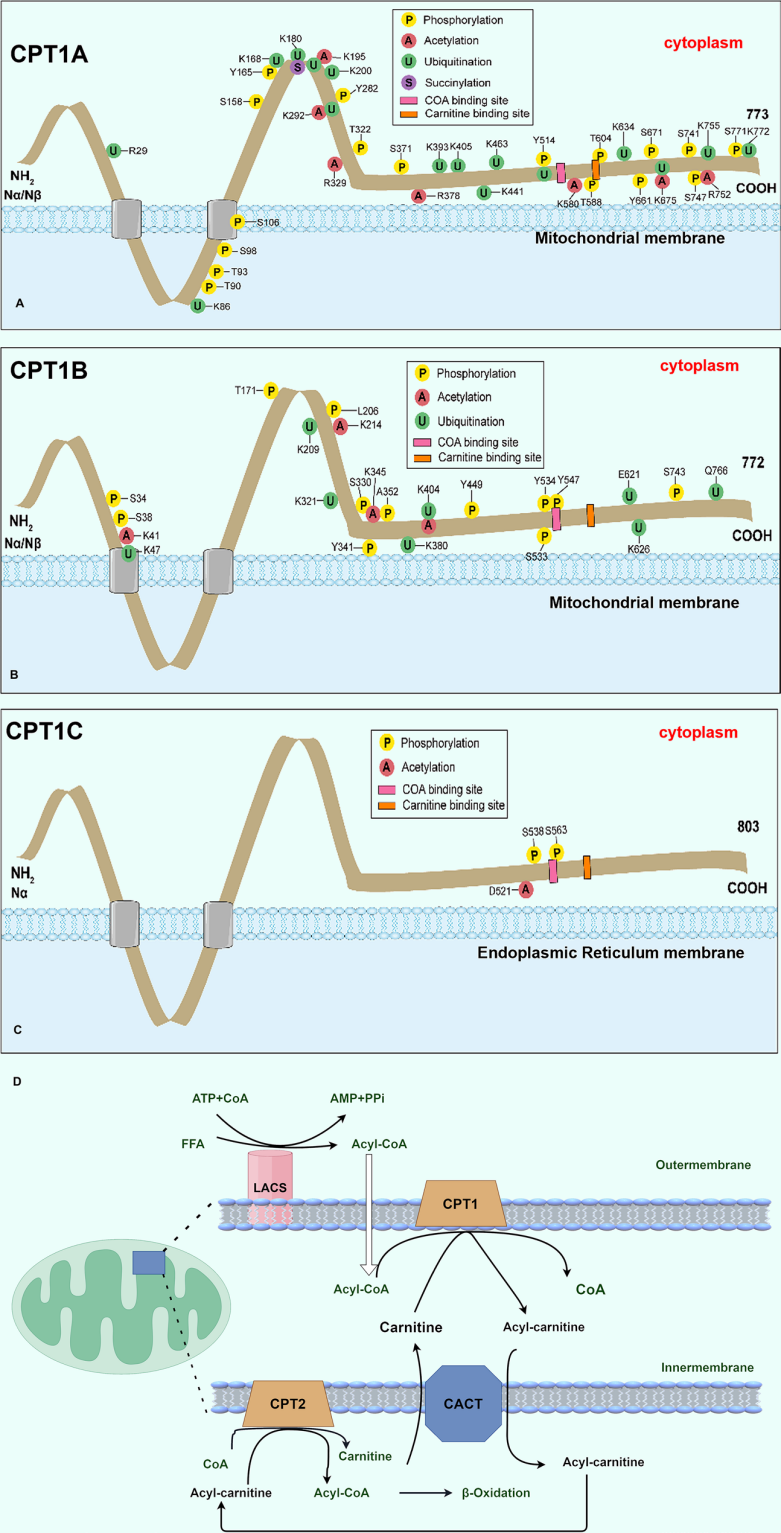
Structural localization and functions of CPT1 genes. **A–C**. The outer mitochondrial membrane is the main site of expression for CPT1A and CPT1B, whereas CPT1C is predominantly localized on the outer surface of the endoplasmic reticulum. **D**. Functions of CPT1. Long-chain fatty acids cannot directly penetrate the inner mitochondrial membrane and rely on CPT1 to initiate the “carnitine cycle” for transport. The steps can be broadly divided into catalyzing key reactions, assisting transmembrane transport, and supporting subsequent oxidation

CPT1A and CPT1B are anchored to the inner surface of the outer mitochondrial membrane, each consisting of two transmembrane structures facilitating signal transduction, protein transport, and enzymatic reactions. They also have a short linker loop maintaining their structural stability. The short N-terminal hydrophobic region of the peptide chain and the long C-terminal region have distinct roles: the N-terminal is regulated by malonyl-CoA (MCoA), and the C-terminal binds with carnitine to exert catalytic functions [11] (Figs. 2A–C).

CPT1A and CPT1B are widely expressed, catalyzing the conversion of long-chain acyl-CoA and carnitine to acylcarnitine, which enhances β-oxidation and ATP release. CPT1C is predominantly expressed in neurons, has weaker catalytic activity, and primarily contributes to energy supply and neural regulation. Post-translational modifications further diversify CPT1 functions (Table 1) [12].

**Table 1:** Characteristics of CPT1A, CPT1B and CPT1C

Characteristic	CPT1A	CPT1B	CPT1C
Amino acid quantity	773	772	803
Molecular weight (kDa)	88	37	91
Organelle distribution	Mitochondria	Mitochondria	Endoplasmic reticulum
Tissue distribution	Liver, kidney, breast	Skeletal muscle, heart	Brain
Functions	Long-chain FAO, nucleotide synthesis	Long-chain FAO, nucleotide synthesis	Nutrient delivery, regulatory activity
Substrate	Carnitine, acyl-CoA carnitine	Carnitine, acyl-CoA carnitine	Uncertain
Catalytic activity	Strongest	Strong	Weak or absent
Chromosomal location	11q13.3	22q13.3	19q13.3

### Functions

Long-chain fatty acids ( > 12 carbons) differ from medium- and short-chain fatty acids in that they cannot directly cross the mitochondrial membrane. They are first converted to acyl-CoA and subsequently transported into mitochondria by CPT1 (Fig. 2D).

CPT1A and CPT1B catalyze the conjugation of long-chain acyl-CoA with carnitine to form acylcarnitine, which is translocated across the inner mitochondrial membrane by carnitine–acylcarnitine translocase. CPT2 then regenerates acyl-CoA in the mitochondrial matrix, releasing carnitine to the intermembrane space via carnitine–acylcarnitine translocase. Acyl-CoA undergoes β-oxidation, producing acetyl-CoA, which subsequently enters the tricarboxylic acid cycle to generate CO_2_, NADH, and FADH2. These reducing equivalents fuel the mitochondrial respiratory chain to drive ATP synthesis. CPT1C, on the other hand, is largely excluded from the carnitine cycle and functions primarily in the central nervous system, where it regulates neuronal energy supply and signaling. In cancer, CPT1C may act as a metabolic sensor or modulator of cellular senescence [13].

### Cellular physiological processes involving CPT1

CPT1 is essential for normal physiology. Its deficiency induces hepatic dysfunction in mice on high-fat diets [14]. S-1-propionylcysteine enhances FAO by upregulating CPT-1 activity and reducing MCoA levels in cardiac and skeletal muscle, improving endurance performance [15]. Further, Su et al. reported a reduction in FAO and CPT1A expression in mesothelial cells from patients on long-term peritoneal dialysis and suggested FAO enhancement in these cells as a potential treatment for peritoneal fibrosis [16]. Besides, high CPT1B expression in cardiomyocytes disrupts long-chain fatty acid metabolism and exacerbates cardiac defects when the heart encounters ischemia or failure due to loss of connection with the cellular oxygen sensor PHD2/3 [17]. Mutations in CPT1A may result in fatty acid metabolism disorders and hereditary diseases, such as primary carnitine deficiency, while mutations in CPT1C can cause neurological diseases, including hereditary spastic paraplegia [18].

### Abnormal CPT1 expression in disease

CPT1 dysregulation is implicated in multiple diseases. In metabolic-associated steatotic liver disease, CPT1 expression is downregulated, promoting fatty liver pathology [19]. For example, butyl phthalate exposure reduces CPT1A expression in young mice, causing steatosis, while in older mice, it induces fibrosis [20]. The lipid-lowering agent phenoxylic acid activates AMP-activated protein kinase (AMPK), thereby upregulating CPT1 and improving metabolic-associated steatotic liver disease outcomes [21]. In hepatocellular carcinoma, particularly during cachexia, CPT1B dysregulation exacerbates metabolic imbalance [22]. In addition, abnormal CPT1A activation is associated with altered oral microbiota in patients with oral cancer [23].

## Regulation of CPT1 expression

### Regulation of CPT1 mRNA levels

Peroxisome proliferator-activated receptors (PPARs) are ligand-dependent transcriptional factors that regulate CPT1 gene expression via PPAR response elements [24]. The three PPAR subtypes—PPARα, PPARβ/δ, and PPARγ—regulate CPT1 gene expression, as demonstrated in various in vivo models. PPARα enhances CPT1A transcription in fatty liver by promoting mitochondrial fusion protein 2 expression and restoring lipid metabolism [25, 26]. CPT1B binds to the transcription factors myocyte enhancer factor 2A and myocyte enhancer factor 2C in skeletal muscle, synergizing with PPARα activity [27]. In contrast, PPARα inhibition downregulates CPT1A expression and reduces inflammatory bowel disease incidence [28]. In mouse aortic endothelial cells, PPARβ/δ influences CPT1 to block lipid-induced increases in reactive oxygen species (ROS) levels and decrease nitric oxide bioavailability [29]. PPARγ, an enhancer of regulatory T cell-related immune responses, has been found to upregulate CD36 and CPT1 expression to alleviate autoimmune diseases [30]. In hepatocellular carcinoma, PPARγ-specific activation of RNF5 facilitates K63-linked ubiquitination of IGF2BP1, increasing CPT1A expression and accelerating tumor progression [31].

Non-coding RNAs also regulate CPT1. Circular RNAs sequester miR-106a-5p and miR-320a, indirectly suppressing CPT1 expression and slowing nonalcoholic fatty liver disease progression [32]. Downregulation of has-miR-124-3p, has-miR-129-5p, and has-miR-378 expression enhances carnitine–acylcarnitine transporter activity in prostate cancer, increasing fatty acid uptake and metastasis [33]. Similarly, estrogen-related receptor α, miR-1291, and CPT1C collectively regulate cancer cell proliferation, energy metabolism, and oncogenic transformation [34]. In hepatocellular carcinoma (HepG2 cells), miR-370 directly downregulates CPT1A expression at the transcriptional level, reducing FAO rates and promoting lipid accumulation [35]. Transfer RNA-derived fragment-16 suppresses lung cancer progression by impairing IGF2BP1 binding to CPT1A via N6-methyladenosine modification, thereby destabilizing CPT1A transcripts [36].

**Fig. 3:**
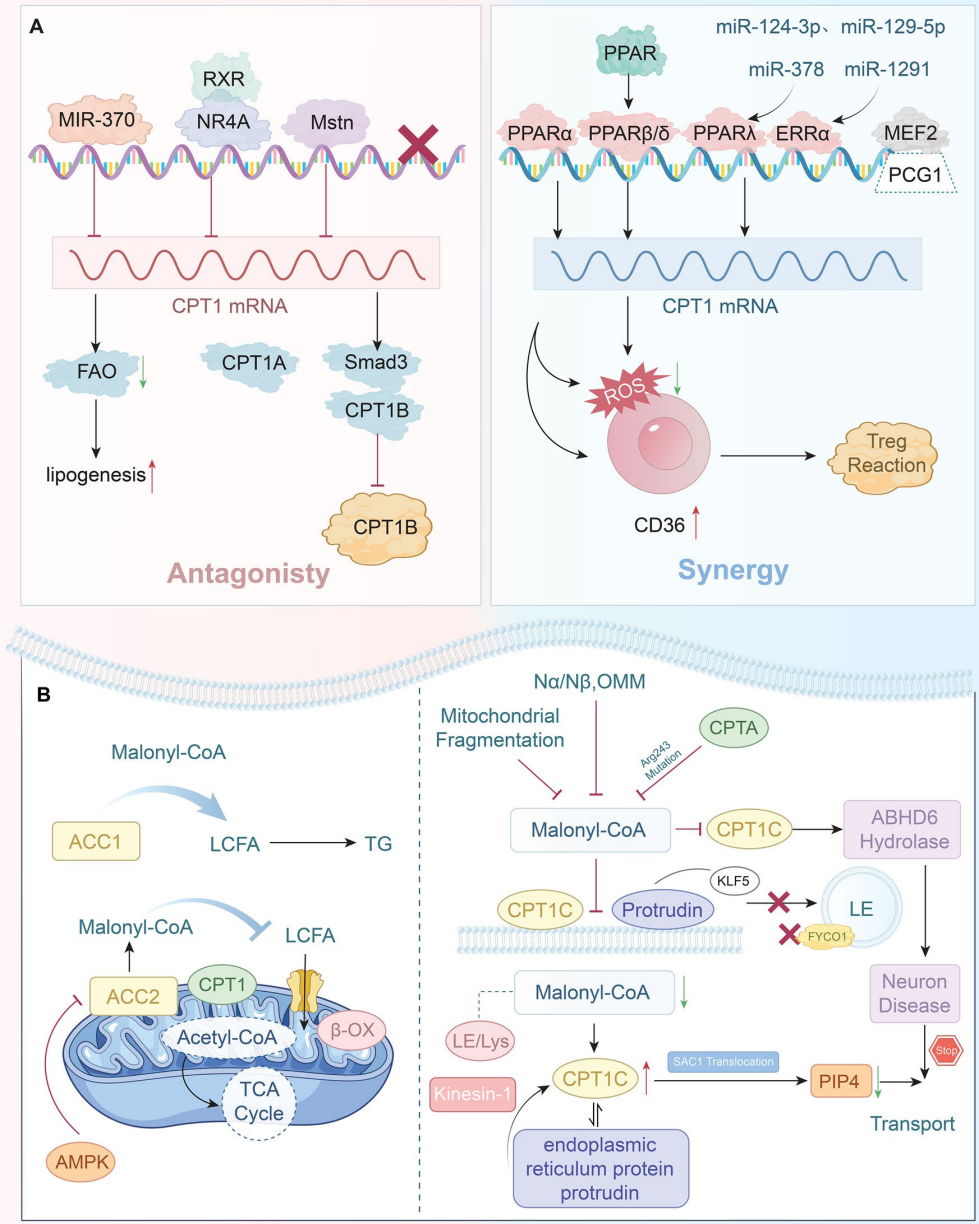
CPT1 is regulated at both the mRNA and protein levels. **A**. CPT1 is regulated by both negative and positive mechanisms at the mRNA level. **B**. Multiple mechanisms by which malonyl-CoA regulates CPT1 protein activity

Additional mechanisms of CPT1 regulation include its modulation by nuclear receptors. Retinoid X receptor, a member of the nuclear receptor superfamily, forms dimers with nuclear receptors (Nur77 and NURR1) of the NR4 subfamily, influencing the transcription of tumor suppressor genes and downregulating CPT1A expression [37]. Notably, the myostatin gene inhibits muscle development and enhances fat content. The downstream transcription factor SMAD3 of this gene directly binds to the promoter of the CPT1B gene, downregulating the expression of CPT1B and suppressing the β-oxidation of intramuscular fatty acids [38]. In the case of CPT1C, Yin Yang 1 can directly activate its transcription under hypoxic conditions, affecting pancreatic cancer cell proliferation [39]. Fig. 3A illustrates the negative and positive regulation of CPT1 at the mRNA level.

### Regulation of CPT1 protein expression levels

MCoA, a product of fatty acid synthesis, inhibits CPT1 protein activity through negative feedback, limiting acylcarnitine input. Intriguingly, although CPT1A and CPT1B are highly homologous, CPT1A is more susceptible to MCoA. Despite CPT1C being exclusively expressed in the brain, it exhibits a high affinity for MCoA. MCoA activity is closely associated with CPT1 expression and influenced by changes in mitochondrial function.

First, mitochondrial fragmentation in response to external stimuli reduces the inhibitory capacity of MCoA on CPT1, thus enhancing FAO, stimulating gluconeogenesis, and inducing insulin secretion in pancreatic β cells to regulate fatty acid utilization [40]. Coarse-grained molecular dynamics studies have demonstrated that MCoA regulation of the N-terminal of CPT1 is affected by the curvature of the outer mitochondrial membrane. This curvature determines whether the N-terminal of CPT1 presents as inhibitory Nα or non-inhibitory Nβ. These distinct manifestations display different susceptibilities to MCoA [41]. Furthermore, in a mouse model, it was indicated that MCoA-dependent inhibition of CPT1B reduced the sensitivity to MCoA, delaying myocardial hypertrophy development [42]. Mutations at the CPT1A site can compromise MCoA activity. Butyrate is transformed into butyryl-CoA via short-chain family member 2 of acyl-CoA synthetase. Hao et al. reported that mutation of CPT1A at Arg243, an amino acid essential for MCoA association, impaired the binding of both MCoA and butyryl-CoA, attenuated induced regulatory T cell generation, and disrupted intestinal immune homeostasis [43]. It has also been reported that elevated glucose levels, such as in hyperglycemia with hyperinsulinemia, increase MCoA levels, deterring CPT1-mediated FAO and promoting glucose utilization [44, 45].

Although CPT1C is not involved in FAO, it provides nutrients for neurotransmitter transmission and axonal growth, which highlights its potential in treating central nervous system diseases. In cortical neurons, glucose consumption reduces MCoA levels, enabling CPT1C to mediate rapid excitatory neurotransmission [46]. Miralpeix et al. reported that MCoA-mediated negative regulation of CPT1C enhances ABHD6 hydrolase activity in the hypothalamus, thereby promoting the progression of neuron-related diseases [47]. Furthermore, inhibition of MCoA synthesis relies on CPT1C to decrease lysosome/late endosome abundance at the axon terminal, shortening axon length [48] (Fig. 3B).

CPT1 activity is also modulated by multiple post-translational modifications, including acetylation, ubiquitination, succinylation, and phosphorylation. CPT1A has been studied most extensively. Protein acetylation is critical for chromatin stability, protein–protein interactions, cell cycle control, cell metabolism, nuclear transport, and actin nucleation. CPT1A shows six acetylation modification sites: K195, K292, R329, R379, K508, and K675. High-fat and high-fructose diets upregulate ketohexokinase-C expression, prompting acetylation at the K508 site, which leads to FAO dysfunction. Ketohexokinase-C overexpression can also reduce CPT1A expression and increase triglyceride accumulation [49]. Succinylation is a process where a succinyl donor covalently attaches a succinyl group to a lysine residue, mediated by succinyl Co-A. This modification is closely associated with, for example, neurological diseases, inflammation, and metabolic diseases. CPT1A utilizes succinyl Co-A as a substrate to succinylate proteins in vitro, and its lysine succinyltransferase activity appears to be independent of its CPTase activity [50]. CPT1A facilitates succinylation at the K302 site to impede Parkin-mediated degradation of mitochondrial fission factor, regulating mitochondrial dynamics and activating sterol regulatory element-binding protein 1 to promote lipid desaturation in ovarian cancer 51, 52]. Similarly, considering the existence of ubiquitination modification sites, CPT1A regulates numerous biological processes, such as protein degradation, DNA damage repair, cell autophagy, endocytosis, and inflammatory responses. Dysfunctions in the ubiquitin pathway can lead to metabolic syndrome and inflammatory diseases. For instance, in lipopolysaccharide-induced sepsis, signal transducer and activator of transcription 3 (STAT3) expression was found to be upregulated, which inhibited the ubiquitination of CPT1A produced by macrophages, stabilizing CPT1A expression under the mediation of USP50. This led to the exacerbation of sepsis and FAO, a symptom alleviated by curcumin [53]. In addition, owing to its succinylation activity, CPT1 affects other proteins as well. In extranodal natural killer/T-cell lymphoma, nasal type, CPT1A promotes the succinylation of lactate dehydrogenase A at K318, and either lactate dehydrogenase A knockdown or K318 mutation (K318R) abolishes the oncogenic effects of CPT1A [54]. CPT1A-mediated succinylation of SP5 induces transcription of 3-phosphoinositide-dependent protein kinase 1, activating the AKT/mTOR signaling pathway in prostate cancer [55].

**Fig. 4:**
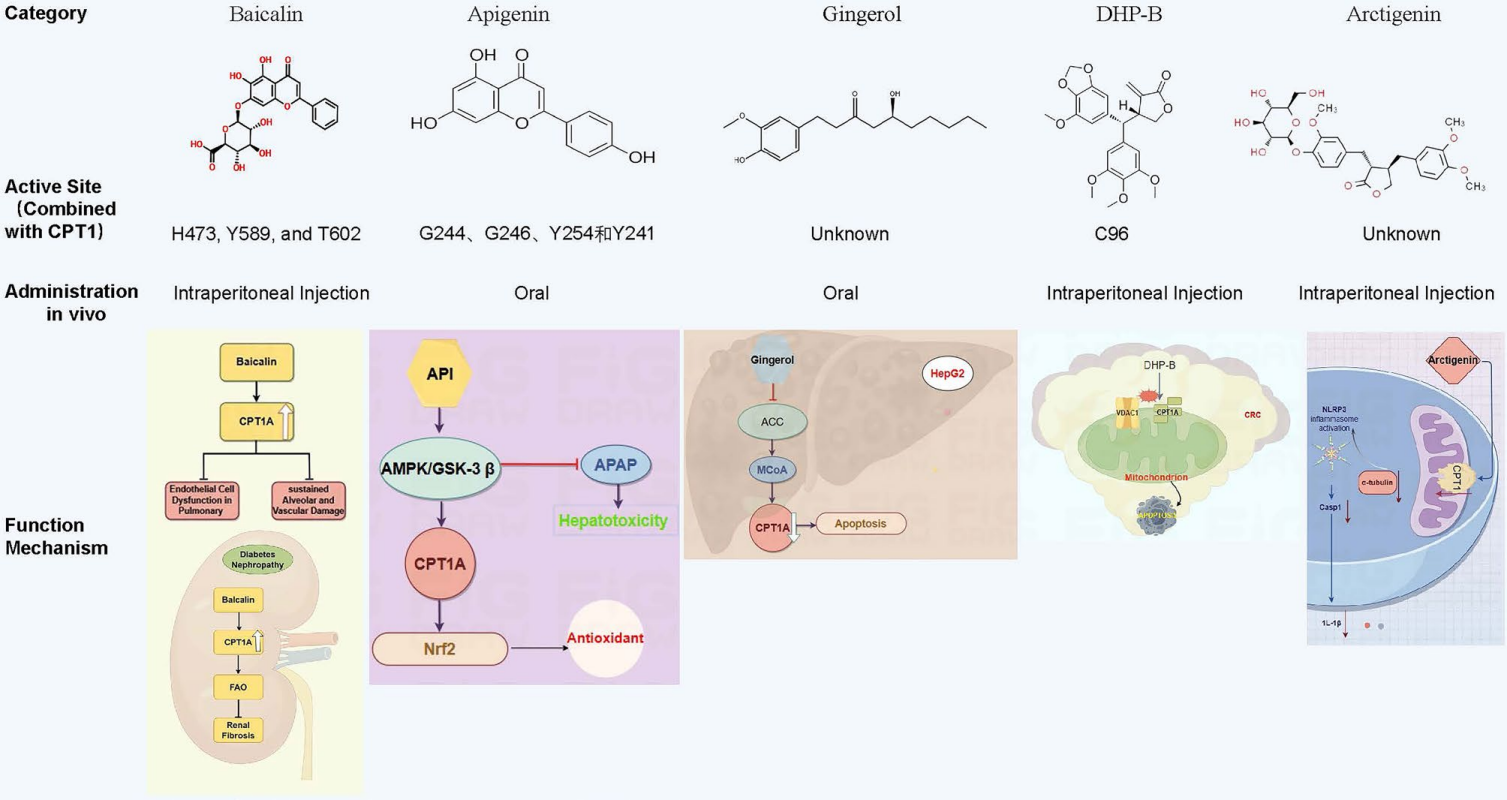
Regulation of CPT1 protein activity by five representative natural compounds

Natural compounds can also modulate CPT1 activity (Fig. 4). Baicalin, a flavonoid from *Scutellaria baicalensis* roots, has been found to upregulate CPT1A expression to ameliorate pulmonary endothelial dysfunction under hyperoxia [56] and attenuate renal fibrosis in diabetic kidney disease [57]. Apigenin enhances CPT1A activity via the AMPK/GSK-3β pathway, potentially mitigating drug-induced liver damage [58]. In contrast, gingerol derivatives decrease CPT1 expression by inhibiting acetyl-CoA carboxylase, elevating MCoA levels [59]. In colorectal carcinoma, 2,6-dihydroxypeperomin B from *Peperomia dindygulensis* covalently binds CPT1A at Cys96, blocking CPT1A–VDAC1 interaction to promote apoptosis [60]. *Arctium lappa* extracts downregulate CPT1 expression in macrophages, limiting FAO, reducing α-tubulin acetylation, and preventing NLRP3 inflammasome assembly, ultimately mitigating colitis [61]. Parthenolide, a bioactive compound from feverfew shoots, exhibits antitumor activity by inhibiting FAO [62].

Some fatty acid metabolism-related genes, such as CD36 and fatty acid binding protein 3 (FABP3), also influence cellular function via CPT1 interactions. Table 2 summarizes eight representative genes and their functional roles [63–70].

**Table 2:** Genes showing protein–protein interactions with CPT1

Factor	Description	Cellular function	Disease type	Target
CD36	Fatty acid translocase	Promotes fatty acid uptake, β-oxidation	Infection	CPT1A
FABP3	Fatty acid binding protein 3	Mediates fatty acid transport	Sarcopenia	CPT1A
PDK4	Pyruvate dehydrogenase kinase 4	Inhibits CPT1A activity	Thyroid disease	CPT1A
PLIN5	Perilipin 5	Regulates FAO	Cardiomyopathy	CPT1
ACSL	Acyl-CoA synthetase long chain	Activates long-chain fatty acids to fatty acyl-CoA	Various cancers	CPT1A
FADS	Fatty acid desaturase	Fatty acid biosynthesis and metabolism	Obesity	CPT1A
SCD	Stearoyl-CoA desaturase	Generates monounsaturated fatty acids	Obesity	CPT1
LPL	Lipoprotein lipase	Hydrolyzes triglycerides	Nonalcoholic steatohepatitis	CPT1

## Role and functions of CPT1 in cancer

Recent evidence, although still emerging, indicates that malignant cells can reprogram lipid metabolism to support tumor growth, proliferation, invasion, metastasis, and immune evasion. Understanding these mechanisms is thus critical for elucidating the oncogenic role of CPT1.

### CPT1 promotes tumor cell proliferation

Adipocytes are key regulators of tumor cell proliferation. In prostate cancer, CPT1 is regulated by the fatty acyl-CoA synthase ASCL1, which promotes triglyceride accumulation and enhances tumor growth in mice [71,72]. In breast cancer, adipocytes release metabolic substrates, supporting tumor proliferation and reducing the efficacy of HER2-targeted therapies [73–75].

Ras pathway activation, exacerbated by non-alcoholic hepatic steatosis, accelerates genomic damage and promotes hepatic carcinogenesis. CPT1A inhibition using etomoxir (ETO) suppresses Ras-dependent liver tumor growth [76]. Interestingly, feedback upregulation of CPT1A can partially counteract anticancer effects caused by SOAT1 deficiency [77]. Adipocytes also secrete adipokines, such as leptin, adiponectin, acylation-stimulating protein, omentin, and cytokines. Dysbiosis alters adipokine synthesis and secretion. In colorectal cancer murine models on high-fat diets, enrichment of *Corynebacterium* ST1911 activates CPT1A and downstream extracellular signal-regulated kinase (ERK) signaling, promoting malignant progression and disrupting lipid homeostasis [78]. Fasting-mimicking diets have been shown to counteract uncontrolled tumor growth. Single-cell RNA sequencing of intratumoral immune cells has revealed that this diet induces RUNX3 acetylation, inactivating CPT1A, reducing B cell to IgA conversion, and promoting tumor regression [79]. Lipid droplets buffer fatty acids derived from autophagy, protecting mitochondria from lipotoxicity in colorectal cancer cells under acidic microenvironments. Inhibiting lipid droplet biogenesis causes mitochondrial dysfunction, which can be rescued via CPT1A inhibition [80].

Amino acid metabolic disorders also indirectly influence tumor outcomes. Normal catabolism of branched-chain amino acids, such as valine, leucine, and isoleucine, produces short-chain fatty acids, which are exported from mitochondria as short-chain carnitines. Metabolic disorders disrupt CPT1A, preventing long-chain carnitine transport, promoting triglyceride storage as lipid droplets, hindering FAO, and ultimately limiting pancreatic ductal adenocarcinoma growth [81]. Lipid droplet formation can be regulated by hypoxia-inducible factor; for example, MED15-mediated CPT1A activation has been reported to drive hypoxic tumor progression [82], and lipid droplet–mitochondria interactions mediated by PLIN2, a lipid differentiation-related protein, and CPT1A via phosphofructokinase, liver type promote lipid mobilization [83, 84]. Moreover, the overexpression of leucine-rich repeat kinase 2 facilitates β-oxidation in HepG2 cells and positively affects CPT1A at the transcriptional level by activating AMPK and PPARα, driving cancer cell proliferation [85]. Several studies have shown that valine-containing protein in colorectal cancer binds to histone deacetylase 1, promoting the degradation of valine-containing protein, activating the transcription of CPT1A, and further promoting the growth of tumors [86]. Under glutamine deprivation, CPT1A enzymatic activity is maintained through interaction with tripartite motif-containing protein 2, protecting cells from apoptosis [87].

**Fig. 5:**
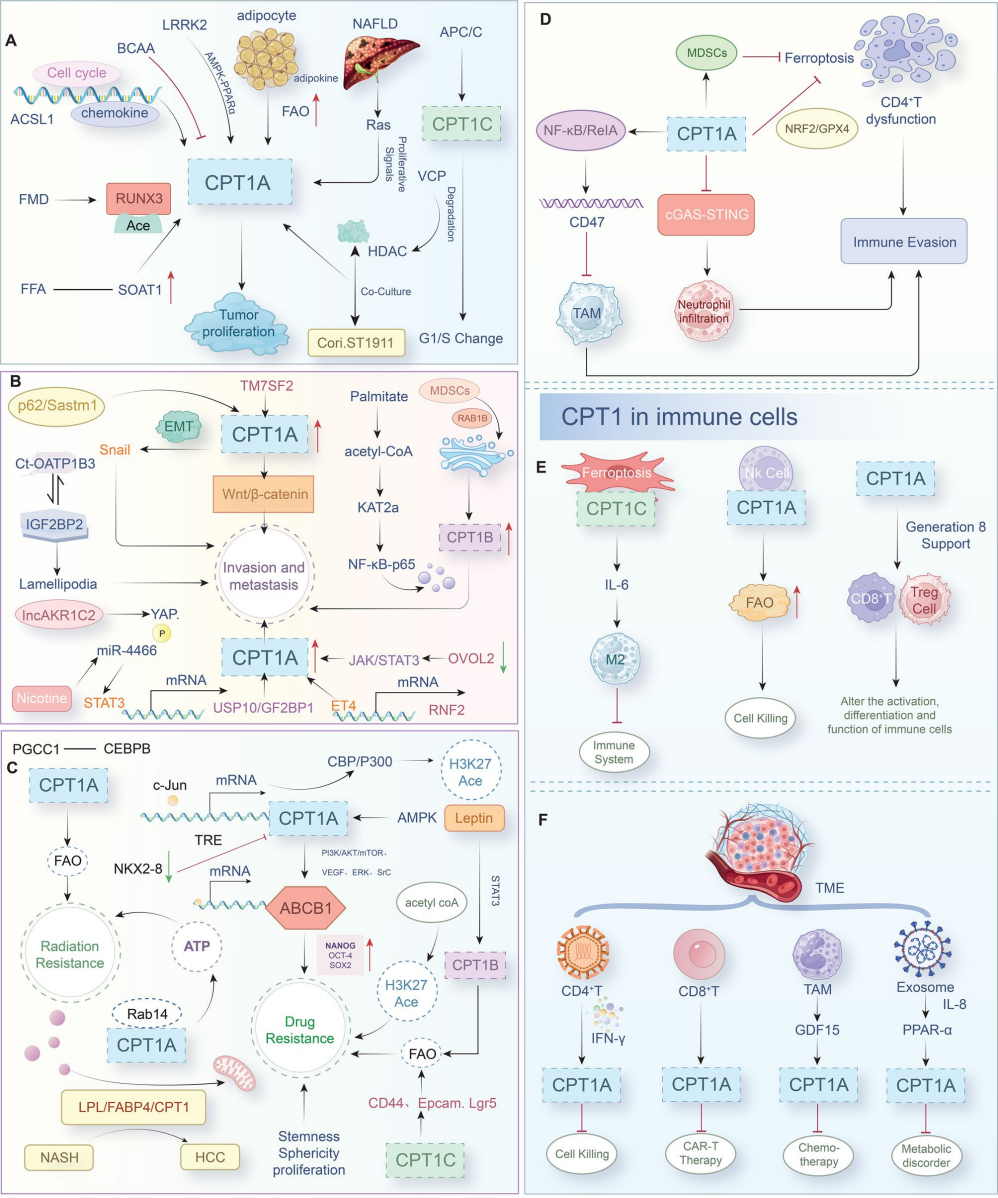
Role of CPT1 in tumor proliferation, invasion, and metastasis; therapy resistance; and immune evasion **A**. CPT1 promotes uncontrolled tumor cell proliferation. **B**. CPT1 is closely associated with tumor metastasis and invasion. **C**. CPT1 contributes to radiotherapy and chemotherapy resistance. **D**. CPT1 in tumor cells influences the TME. **E**. CPT1 regulates immune cell function within the TME. **F**. TME regulates CPT1 expression in tumor cells

Epigenetic modifications are also involved in the regulatory role of CPT1A on tumors. Bromodomain and extraterminal domain (BET) proteins are epigenetic readers that control oncoprotein expression. The antiproliferative effect of BET inhibitors benefits from altering mitochondrial dynamics, amplifying ATGL expression and lipase activity, and downregulating CPT1A expression to induce cell cycle arrest and cell death [88]. In contrast, CPT1C, regulated by APC/C, promotes esophageal squamous cell carcinoma cell survival in harsh metabolic environments by upregulating energy supply and accelerating the G1/S transition [89]. Therefore, tumor cell growth and reproduction depend on the cell cycle, CPT1 substrate concentration, and lipid homeostasis (Fig. 5A). Targeting the CPT1A expression pathway seems like a promising cancer therapeutic strategy.

### CPT1 promotes tumor metastasis and invasion

Tumor metastasis is a critical step in the malignant progression of cancer. Cancer cells undergo three major stages: invasion, circulation, and colonization. These are essential to complete metastasis, which involves releasing proteases to destroy adjacent basement membranes and extracellular matrices. This enables tissue invasion and microcirculatory penetration, followed by entry into the blood and lymphatic circulation, evasion of immune surveillance, and eventual colonization in the microenvironment of distant organs.

CPT1 has been confirmed to endow cancer cells with metastatic potential. The emergence of pseudopodia is necessary for tumor cell movement. Continuous CPT1A transcription is jointly regulated by cancer-type organic anion transporting polypeptide 1B3 and insulin-like growth factor 2 mRNA-binding protein 2, and its function is related to lamellipodia formation in tumor cells [90]. Epithelial–mesenchymal transition (EMT) also mediates metastasis. The Wnt/β-catenin/EMT axis indicates Wnt as an important upstream regulator of EMT. Liu et al. found that TM7SF2-induced lipid reprogramming promotes cervical cancer migration through the CPT1A/Wnt/β-catenin axis [91]. Snail stability in EMT, regulated by CPT1A, can also promote pancreatic adenocarcinoma cell migration [92]. Deficiency of the epithelial transcription factor ovo-like 2 has been reported to downregulate CPT1A expression via JAK/STAT3, weakening lung metastasis [93]. Cancer cell metastasis relies not only on the cells themselves but also on the metastatic niche. Breast cancer cells depend on CPT1A for FAO and, in turn, activate NF-κB signaling in a lysine acetyltransferase 2a-dependent manner through p65 acetylation, conferring metastatic characteristics [94]. In contrast, increased expression of the ETS variant transcription factor 4 and decreased expression of the ubiquitinase ring finger protein 2 inhibit CPT1A expression, preventing esophageal squamous cell carcinoma cells from using anchorage-independent growth of the metastatic niche, affecting tumor invasion ability and inhibiting metastasis [95]. CPT1A-mediated ROS elimination can also sometimes increase metastatic ability by inhibiting tumor anoikis [96]. Some metastasis signaling pathways are also affected by proteins secreted by the tumor. For example, osteopontin, a secreted phosphorylated glycoprotein, interacts with its receptors integrin and CD44, regulating tumor invasion and metastasis. Selective inactivation of the signal transduction adapter p62/Sqstm1 in adipocytes, secretion of osteopontin, and upregulation of CPT1A expression drive prostate cancer invasion [97]. Phosphatidylinositol transfer protein cytoplasmic 1 has been found to recruit RAB1B to the Golgi apparatus and upregulate the expression of CD36 and CPT1B to achieve gastric cancer omental metastasis [98]. Epigenetic reprogramming is a hallmark of breast cancer cells with metastatic ability. m6A modification plays a key role in cancer metastasis. Overexpression of the m6A reader IGF2BP1 is associated with breast cancer metastasis. IGF2BP1 directly recognizes and binds to the m6A site on CPT1A mRNA, increasing its stability and mediating IGF2BP1-induced breast cancer metastasis; this mechanism has been verified in clinical samples [99].

CPT1 also provides metabolic energy for invasion. In esophageal carcinoma, PKN2 expressed in polymorphonuclear myeloid-derived suppressor cells enhances immunosuppressive activity via STAT3 phosphorylation and CPT1B expression upregulation, resulting in infiltration and subsequent tumor proliferation [100]. Sorafenib treatment suppresses CPT1A stability, with overexpression of angiopoietin-like protein 3 strongly inhibiting cell motility [101].

Extracellular vesicles and microRNAs are essential mediators of metastatic spread. Exosomes, which are 30–150 nm vesicles involved in intracellular communication, transport proteins, lipids, and non-coding RNAs that regulate tumor progression. In gastric cancer, exosomal lncAKR1C2 has been reported to suppress YAP phosphorylation, upregulating CPT1A expression and promoting lymphatic metastasis [102]. Likewise, in gastric carcinoma, extracellular vesicles reprogram bone marrow mesenchymal stem cells via the ERK–PPARγ axis to upregulate CPT1A expression and support metastasis [103]. Chronic nicotine exposure enhances brain tumor metastasis through exosomal STAT3 activation, miR-4466 secretion, and SKI/SOX2-mediated CPT1A expression upregulation [104]. Furthermore, miR-328-3p, miR-33b, and miR-365-3p have been found to exert diverse, context-dependent roles in metastasis [105–107]. Thus, tumor metastasis is orchestrated by both intrinsic oncogenic programs and extrinsic cell–cell signaling. Positioned as a metabolic “control center,” CPT1 regulates key steps of the metastatic cascade, representing a compelling therapeutic target (Fig. 5B).

### CPT1 heightens radiotherapy and chemotherapy tolerance

Resistance to radiotherapy and chemotherapy remains a significant challenge in cancer treatment. Drug-resistant tumor cells often display a metabolic shift toward FAO, with sensitivity to therapy closely linked to the lipid composition of cell membranes. Factors such as glycerophospholipid, cholesterol, and sphingomyelin content; the degree of fatty acid unsaturation; membrane fluidity; oxidative stress resistance all influence therapeutic response.

Tumor stemness guided by CPT1 promotes chemotherapy tolerance. Yang et al. reported that the targeted inhibition of the LPL/FABP4/CPT1 fatty acid metabolic axis can effectively prevent the progression of nonalcoholic steatohepatitis to liver cancer [70]. In breast cancer, FABP4 exhibits strong affinity for long-chain fatty acids and enhances lipid uptake and metabolism through CPT1B [108]. Lymph node metastatic cervical cancer cells utilize FAO-derived acetyl Co-A to increase histone H3K27 acetylation at the promoters of stemness genes, as well as enhance the expression of pluripotency-related transcription factors (SOX2, OCT4, and NANOG) and the cervical cancer stem cell marker CD44 [109]. In addition, CPT1C promotes gastric cancer drug resistance by enhancing FAO and subsequently upregulating the mRNA expression levels of gastric cancer stem cell markers (CD44, EpCam, and Lgr5) [110].

CPT1 also augments chemotherapy resistance by enhancing drug efflux. Resistant cells exhibit increased uptake of fatty acids, upregulation of ABC transporter expression, and activation of drug resistance-related pathways (e.g., PI3K/AKT/mTOR, VEGF, ERK, and Src pathways). Acquired paclitaxel resistance in ovarian cancer is associated with the synergistic actions of P-glycoprotein and multiple basic biological processes. Fatty acid synthase and stearoyl-CoA desaturase are central to *de novo* fatty acid synthesis and can reverse paclitaxel resistance by targeting the upstream promoter region of CPT1A to inhibit its transcription [111]. In chemotherapy-resistant cells, estrogen-related receptor γ directly binds to the promoter of ABCB1 to enhance its transcription and simultaneously regulates CPT1B, thereby promoting FAO [112]. In estrogen receptor-positive breast cancer, SLC31A1-mediated copper transport drives CPT1A expression, and SLC31A1 knockout restores tamoxifen sensitivity [113]. Pharmacological inhibition or CRISPR knockout of CPT1A can reverse carboplatin resistance in high-grade serous ovarian cancer cells, as verified in both drug-resistant and sensitive patient-derived xenograft models [114]. In advanced non-small cell lung cancer, CPT1C modulates MRP1 expression under the regulation of E3 ubiquitin ligase NEDD4 [115].

(3) CPT1 and adipose microenvironment-induced drug resistance

The adipose tumor microenvironment (TME) facilitates complex metabolic “dialogues” between adipose tissue and tumor cells through fatty acid metabolism, thereby activating certain drug resistance pathways. This phenomenon is particularly evident in adipose-rich cancers, such as ovarian, breast, and prostate cancer. Feng et al. emphasized that CPT1A interferes with the efficacy of targeted therapies in ovarian cancer through the arginine succinate synthase 1–CPT1A axis [116]. In estrogen receptor-positive breast cancer cells, activated c-Jun recruits CBP/P300 to chromatin, catalyzing histone H3K27 acetylation to increase chromatin accessibility and eliminate the cytotoxic effects of tamoxifen [117]. Leptin secreted by breast adipocytes has been shown to upregulate CPT1B expression in STAT3-induced breast cancer cells [118]. Moreover, CPT1A supports castration-resistant prostate cancer by providing acetyl groups for histone acetylation, promoting growth and anti-androgen resistance [119]. NK2 homeobox 8 inhibits FAO by recruiting the Sin3A/HDAC1/SAP18 transcriptional repression complex. In contrast, the deletion of NK2 homeobox 8 enhances FAO activity, significantly increasing the enrichment of H3K27 acetylation on the CPT1A promoter, leading to fatty acid metabolic reprogramming in epithelial ovarian cancer cells and platinum resistance [120].

Resistance to radiotherapy is also linked to CPT1. PPARγ coactivator-1α forms a complex with CCAAT/enhancer-binding protein β to promote CPT1A transcription, driving CPT1A overexpression and radioresistance in nasopharyngeal carcinoma [121]. FAO itself is a defining feature of radioresistant nasopharyngeal carcinoma. For instance, Rab14 interacts with CPT1A to facilitate fatty acid transport, further contributing to radiation resistance [122].

Collectively, these findings suggest that CPT1 plays a central role in both radiotherapy and chemotherapy resistance (Fig. 5C). Accordingly, pharmacological inhibition of CPT1 appears to be a promising strategy to enhance therapeutic efficacy.

### Role of CPT1 in regulating the immune microenvironment and interfering with immune evasion

Abnormal lipid metabolism within the TME plays a pivotal role in immune evasion. Alterations in lipid metabolism affect not only the energy supply and biosynthetic requirements of cancer cells but also the tumor immune response and therapeutic efficacy. Understanding the role of FAO, and specifically CPT1, in immune cells within the TME is therefore critical for optimizing enhancing antitumor immunotherapy.

CPT1 in tumor cells exerts significant effects on the TME, which comprises stromal, immune, and vascular components. We have found that loss of CPT1A in triple-negative breast cancer (TNBC) activates the cGAS/STING pathway, leading to neutrophil infiltration and promoting an antitumor phenotype [123]. In lung cancer, CPT1A deficiency weakens the immunosuppressive function of myeloid-derived suppressor cells in the TME while enhancing tumor cell ferroptosis [124]. Moreover, CPT1A in lung epithelial cells fosters ferroptosis resistance and CD8^+^ T cell dysfunction in both murine models and clinical settings. L-carnitine produced by tumor-associated macrophages (TAMs) activates CPT1A and c-Myc, upregulates NRF2/GPX4 expression, augments antioxidant capacity, and downregulates ACSL4 expression, thereby suppressing ferroptosis in lung cancer cells. This highlights the therapeutic potential of targeting metabolic crosstalk between TAMs and tumor cells to improve immunotherapy effects [125]. In glioblastoma, CPT1A-derived acetylated acetyl-CoA supports NF-κB/RelA acetylation, which drives CD47 transcription and inhibits macrophage phagocytosis, thereby facilitating immune evasion. Notably, ETO combined with anti-CD47 antibody therapy synergistically enhances macrophage-mediated tumor clearance in radiation-resistant glioblastoma [126] (Fig. 5D).

CPT1 also influences immune cell metabolism and differentiation. For instance, cancer-associated fibroblasts expressing CPT1C in gastric cancer release IL-6, which promotes immunosuppressive M2 macrophage polarization. Clinical data indicate that gastric cancer patients with high CPT1C+ fibroblast infiltration exhibit reduced responsiveness to immunotherapy [127]. During fasting, elevated CPT1A expression in natural killer cells enhances their antitumor activity. A three-week calorie fasting diet reprograms natural killer cells to utilize fatty acids, activating glucocorticoid receptors when glucose is scarce [128]. According to Patel et al., CPT1 supports long-lived immune subsets, such as T_mem_ and M2 macrophages, by ensuring stable energy supply, while also shaping immune responses through metabolic competition and signaling regulation [129]. These findings underscore the pivotal role of CPT1 in immunotherapy research (Fig. 5E).

Conversely, the TME regulates CPT1 expression in tumor cells through signaling and metabolic stress. For example, in melanoma and prostate cancer cells, interferon-γ secreted by CD8^+^T cells upregulates the expression of CPT1A, thereby promoting resistance to CAR-T cell therapy and contributing to immunotherapy failure [130]. In colorectal cancer, TAM-derived GDF15 increases CPT1A expression, which reduces chemosensitivity [131]. Despite the promising clinical outcomes of immunotherapy, innate or acquired resistance to immunotherapy in cancer remains a significant challenge. CD4^+^ T cell-derived interferon-γ induces CPT1A expression, conferring resistance to immune-mediated cytolytic killing in cancer cells [132, 133]. Exosomes from prostate cancer cells further impair tumor-infiltrating CD8^+^ T cells by releasing IL-8, which activates PPARα in receptor cells and subsequently upregulates CPT1A expression in tumor cells, disrupting energy metabolism [134]. Overall, we report that CPT1 acts as a central mediator of tumor–TME interactions by supporting drug and radiation resistance, modulating immune responses, and driving immune escape (Fig. 5F).

**Fig. 6:**
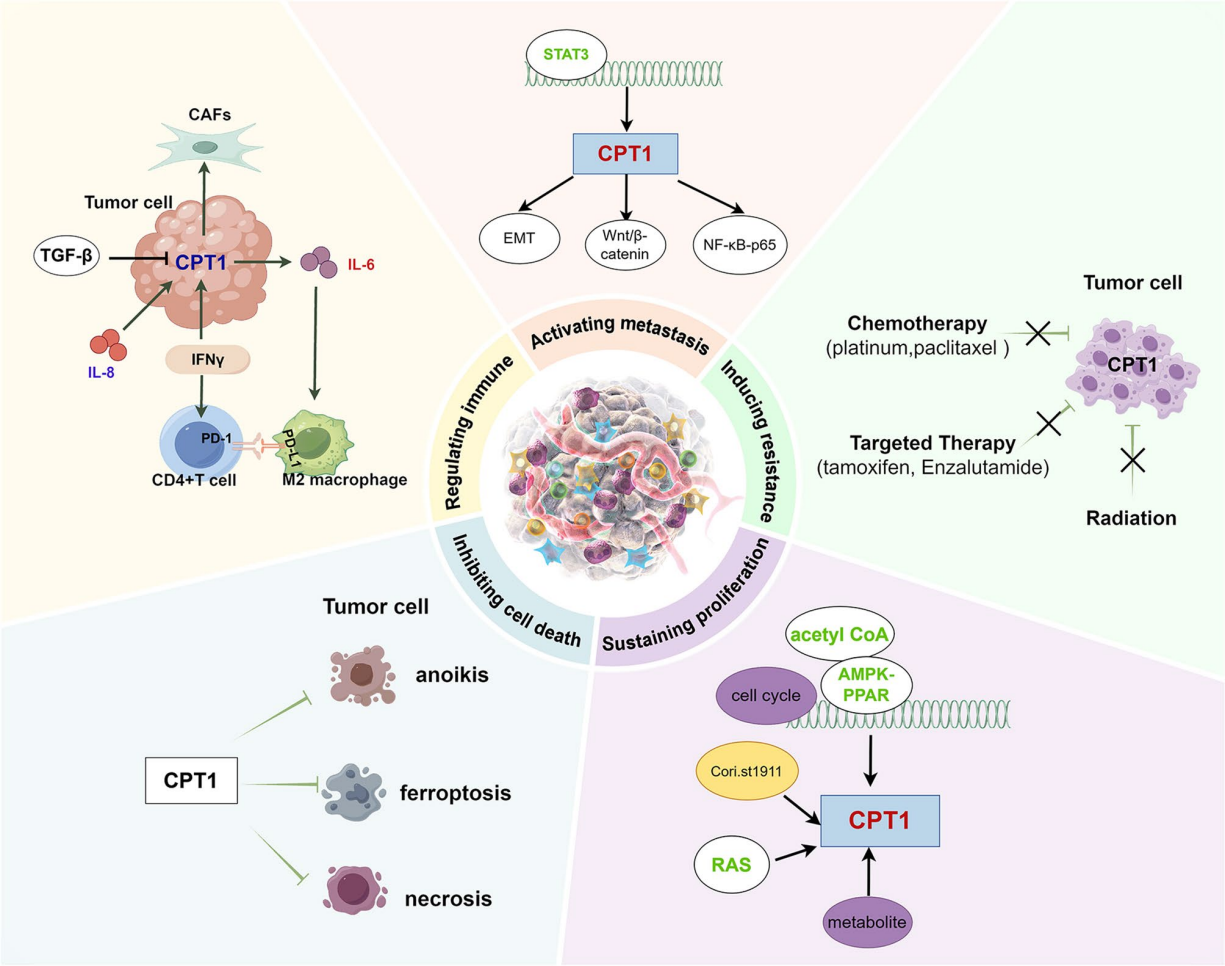
Core regulatory network of CPT1 in relation to tumor progression and therapeutic response. CPT1 exhibits multifaceted roles in tumor proliferation, metastasis, drug resistance, and immune regulation

In summary, CPT1 participates in various tumor-related processes, including continuous proliferation, metastasis, radiotherapy and chemotherapy tolerance, cell death resistance, and immune microenvironment regulation (Fig. 6).

## Targeting CPT1 for cancer therapy

At present, the inhibitors of CPT1 and strategies for their optimization and development remain incomplete. This section summarizes the current progress and future directions of CPT1-targeted cancer therapy.

### Existing CPT1 inhibitors and their antitumor efficacy

Five important CPT1 inhibitors are presented in Table 3. Their specific characteristics are described below. ETO irreversibly binds to the catalytic site of CPT1, suppressing its activity to reduce FAO, lower ATP production, and decrease cancer cell viability [135]. Originally developed for chronic heart failure (to alleviate myocardial pressure load and reduce fatty acid accumulation), ETO has shown promising antitumor potential in combination therapies. For example, in glioblastoma, the combination of ETO and temozolomide (a standard chemotherapeutic) inhibits tumor invasion [136], while in TNBC, ETO synergizes with EZH2 inhibitors to significantly enhance therapeutic efficacy [137]. However, clinical use of ETO is limited by hepatotoxicity, possibly linked to oxidative stress [138], highlighting the need for careful dose optimization and patient selection.

**Table 3:** Functions and applications of CPT1 inhibitor

	Etomoxir	Teglicar (ST1326)	Perhexiline	Oxfenicine
Clinical application	Cardiovascular disease, diabetes, obesity	Type 2 diabetes, leukemia	Angina pectoris, heart failure	Ischemic cardiomyopathy
Side effects	Myocardial hypertrophy, liver injury	Dizziness	Gastrointestinal reactions, abnormal liver function	Gastrointestinal reactions, abnormal liver function
Mechanism	Inhibits CPT-1 catalytic site	Induces apoptosis by sensitizing BCL-2	Inhibits tumor progression via PPAR-γ	Prevents long-chain fatty acid transport
Major toxicities	Hepatic, cardiac	Cellular	Nervous system, hepatic	Hepatic, cardiac

s

ST1326 (teglicar) is a preclinical-stage CPT1 inhibitor with demonstrated antitumor effects, particularly when combined with Bcl-2 inhibitors (e.g., ABT199) in acute myeloid leukemia (AML) [139–142]. ST1326 sensitizes cancer cells to Bcl-2 inhibition, upregulates caspase-9, −8, and −3 mRNA and protein levels, and induces apoptosis; these effects have also been confirmed in canine cancer cell models. However, ST1326 faces a major challenge: cardiotoxicity. Because CPT1B (a CPT1 homolog) is highly expressed in cardiomyocytes, ST1326-mediated inhibition of CPT1B triggers ROS overproduction and free radical accumulation, leading to cardiomyocyte injury. This cardiotoxicity has prevented ST1326 from advancing to late-stage clinical trials, despite initial plans for phase II testing.

Perhexiline, initially developed as an anti-angina drug, also demonstrates anticancer activity. At micromolar concentrations, it inhibits tumor growth and induces apoptosis [143, 144]. Oxfenicine, another CPT1 inhibitor, was originally used to reduce oxygen demand in ischemic cardiomyopathy and to enhance carbohydrate utilization in the heart [145]. Its mechanism involves preventing CPT1 localization to mitochondria, thereby inhibiting FAO [146]. The biotinylated copolymer CP4 differs from small-molecule inhibitors: it targets CPT1 by restricting FAO output and precisely localizing within lipid metabolism pathways [147]. Its polymer-based structure offers potential advantages in targeted delivery (e.g., accumulation in lipid-rich TMEs), although its in vivo antitumor activity and safety remain to be validated.

### Strategies for optimizing CPT1 inhibitor development

Despite their promising antitumor potential, CPT1 inhibitors face major challenges in clinical translation. These include poor isoform specificity (the CPT1 family has three homologs: CPT1A, CPT1B, and CPT1C, with overlapping tissue expression), high off-target toxicity (e.g., ST1326-induced cardiotoxicity), and limited clinical evidence (most studies are preclinical or involve single-agent therapy). Several strategies have been proposed to address these issues.

First, combination therapies can enhance efficacy and reverse drug resistance. Preclinical studies in murine models have demonstrated that inhibiting CPT1 restores sensitivity to anti-angiogenic drugs in lipid-rich tumors, potentially by disrupting FAO-dependent energy supply in drug-resistant cells [148]. In TNBC, low-dose metformin (a biguanide drug) activates the AMPK–acetyl-CoA carboxylase–FAO pathway while also upregulating Src signaling; combining metformin with Src inhibitors shows synergistic efficacy against metastatic TNBC, providing a framework for integrating CPT1-targeted therapies with metabolic regulators [148].

Second, improving inhibitor specificity via structural optimization appears promising. Structural optimization is essential to overcome the lack of isoform specificity. Advances in structural biology, such as X-ray crystallography, cryo-electron microscopy, and AlphaFold 3, enable high-resolution analysis of CPT1 homologs and their inhibitor binding modes [149, 150]. Identifying isoform-specific residues (e.g., differences between CPT1A and CPT1B) allows for rational drug design with improved selectivity.

Third, chemical property adjustment by modifying lipophilicity, hydrophilicity, and or charge distribution can improve tissue penetration and cellular uptake. This approach may allow selective targeting of CPT1A-rich hepatocellular carcinoma cells or CPT1B-rich cardiomyocytes while minimizing off-target toxicity [151].

Fourth, advanced drug discovery and delivery technologies represent a future trend. Virtual screening and computer-aided design techniques enable the rapid identification of highly specific molecules targeting individual CPT1 isoforms through high-throughput screening of compound libraries, followed by in vitro and in vivo validation to confirm their activity [152]. Further, novel drug delivery systems, such as nanoparticles, polymer micelles, and ligand-modified carriers can improve the targeted delivery of CPT1 inhibitors to tumors (e.g., through folic acid or transferrin receptors overexpressed on cancer cells) [153]. Proteolysis-targeting chimeras represent an emerging targeted degradation strategy. They redirect the ubiquitin–proteasome system to selectively degrade aberrantly expressed CPT1 in tumors, rendering CPT1 a “druggable” target and overcoming limitations of conventional inhibition (e.g., incomplete enzymatic blockade) [154]. Multimodal therapeutic integration is another promising approach. Recent studies suggest that combining CPT1 inhibitors with antibody–drug conjugates, cell therapy, gene therapy, or photodynamic therapy can further enhance target specificity and antitumor efficacy [155].

## Dual role of CPT1 in tumorigenesis

Our long-term observations indicate that CPT1-mediated regulation depends on cellular state and microenvironment. In tumors arising in organs with low lipid content and low FAO activity, CPT1 expression may display opposite regulatory patterns.

For example, a BRAFV600E mutation has been reported to downregulate FAO via monoacylglycerol O-acyltransferase 3, thereby contributing to acquired therapeutic resistance in metastatic colorectal cancer [156]. CPT1A suppresses the FOXM1–SOD1/SOD2/CAT axis, regulates post-irradiation intracellular ROS levels, and enhances radiosensitivity [157].

CPT1A also contributes to immune surveillance by enabling the succinylation of PD-L1 at lysine residues [158], leading to its degradation through the endosome–lysosome pathway and the activation of CD8^+^ T cells. However, CPT1A is not expressed in clear cell renal cell carcinoma owing to its lipid-rich phenotype, where FAO is not a primary metabolic feature [159]. In melanoma, viral infection of mitochondria can induce epigenetic perturbations that upregulate CPT1A expression, enhance mitochondrial antiviral signaling protein palmitoylation, and intensify interferon-I responses. Collectively, these processes strengthen antitumor immune control [160].

## Future research directions for CPT1

An increasing body of evidence indicates that dysregulated energy metabolism may underlie the failure of cancer immunotherapy [161]. At present, CPT1 presents significant research potential in the context of immunotherapy. This can be traced back to hematopoietic stem cells, which exhibit strong fatty acid synthesis capacity, rendering hematopoietic stem cell transplantation an effective treatment for certain malignancies. However, mutations in CPT1A may drive malignant transformation of hematopoietic stem cells [162].

**Fig. 7:**
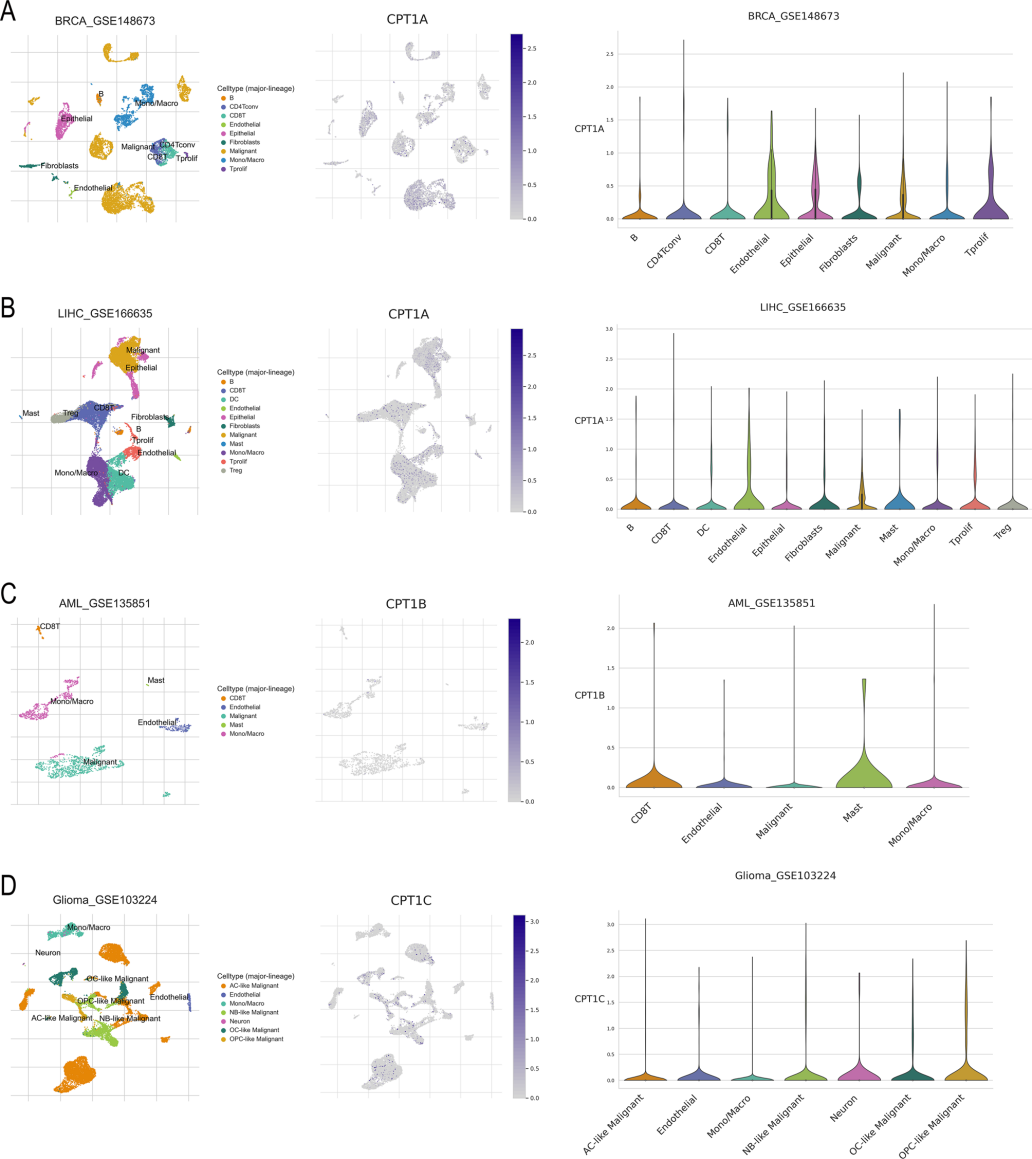
Prediction of CPT1-enriched cell populations at the single-cell level. **A**. CPT1A expression in breast cancer (BRCA_GSE148673) **B**. CPT1A expression in hepatocellular carcinoma (LIHC_GSE166635) **C**. CPT1B expression in AML (AML_GSE135851) **D**. CPT1C expression in glioma (GLIOMA_GSE103224)

The advent of single-cell techniques has enabled researchers to quantify metabolic activity at the level of individual cells within clinical samples, thereby deepening our understanding of the TME and improving diagnostic precision [163]. To assess the role of CPT1 in the TME, single-cell sequencing can be employed to study the clustering and heterogeneity of immune cells. Using the online single-cell analysis database TISCH (http://tisch.comp-genomics.org/), we predicted and characterized CPT1A, CPT1B, and CPT1C expression in different cancer types according to tissue-specific expression patterns (Fig. 7).

In breast cancer (BRCA_GSE148673), 10 cell populations were identified to express CPT1A, with predominant expression in endothelial, epithelial, and proliferating T cells (Fig. 7A). In hepatocellular carcinoma (LIHC_GSE166635), 11 cell populations were detected, with CPT1A expression enriched in endothelial, malignant, and mast cells (Fig. 7B). In AML (AML_GSE135851), five cell populations were identified, with CPT1B predominantly expressed in CD8^+^ T and mast cells (Fig. 7C). In glioma (GLIOMA_GSE103224), seven cell populations were identified, with CPT1C predominantly expressed in nerve cells and oligodendrocyte precursor cells (Fig. 7D).

These findings imply that the three CPT1 homologs exhibit abnormal expression patterns in various immune-related cells. Investigating the reasons behind high CPT1 expression in these cells could provide valuable insights for CPT1-targeted immunotherapy.

## Limitations

There are still limitations in our systematic discussion. This article does not address the compensatory effects among the subtypes of CPT1 due to due to the limited available references. Specifically, it remains unclear whether other subtypes (such as CPT1B) can sustain fatty acid oxidation (FAO) activity through compensatory upregulation when a particular subtype (such as CPT1A) is targeted. Additionally, it is uncertain whether CPT1C influences tumor progression via non-FAO pathways in brain metastases. Furthermore, the impact of co-mutation status in patients (for instance, KRAS and BRAF mutations) on or by CPT1 has not been explored.

## Conclusions and perspectives

Abnormal lipid metabolism remains a significant challenge in the fight against cancer. Despite current therapeutic advances, there is substantial room for improvement. Besides cancer, CPT1 has been implicated in metabolic diseases, such as obesity and diabetes. Therefore, further exploration of novel diagnostic and therapeutic strategies centered around CPT1 is pivotal.

Previous studies have demonstrated that FAO inhibition is an effective strategy to suppress and reverse cancer cell proliferation. However, its impact on immune activation remains insufficiently characterized, as FAO exerts complex, bidirectional regulatory effects on immune cells. The use of humanized murine models has facilitated investigations into the intricate interplay between cancer metabolism and immune responses, enabling researchers to overcome biological variability and develop advanced preclinical tumor models. Moreover, combining machine learning algorithms with single-cell resolution sequencing across spatiotemporal dimensions enables detailed mapping of metabolic processes, cellular differentiation, intercellular communication, signaling pathways, and tumor–microenvironment interactions. This approach offers the potential to refine prognostic assessment systems and deepen understanding of metabolic dynamics at the cellular level.

The characterization of CPT1 as a key FAO enzyme has clarified its roles in tumor proliferation and highlighted its therapeutic relevance. Advances in metabolomic technologies have opened new avenues to probe and modulate the biochemical machinery that drives hypermetabolic, aggressive tumor phenotypes. Recently, innovative therapeutic concepts have emerged to target oncogenic proteins once deemed “undruggable,” including proteolysis-targeting chimeras, CRISPR-based genome editing, RNA-targeting strategies, and biomolecular condensate modulation. Collectively, these insights suggest that metabolic research will enable the rational development of isoform-specific CPT1 inhibitors, facilitating the translation of in vivo proof-of-concept findings into clinically viable therapies. Strategic integration with complementary therapeutic modalities may ultimately enable the design of individualized treatment regimens for patients with cancer, an increasingly feasible goal as research progresses.

## Data Availability

Not applicable.

## Abbreviations

FAO: Fatty acid oxidation
CPT1: Carnitine palmitoyltransferase1
CPT2: Carnitine palmitoyltransferase2
CPT1A: Carnitine palmitoyltransferase1-A
CPT1B: Carnitine palmitoyltransferase1-B
CPT1C: Carnitine palmitoyltransferase1-C
TME: Tumor microenvironment
AMPK: AMP-activated protein kinase
EGFR: Epidermal growth factor receptor
MCoA: Malonyl-CoA
FFA: Free fatty acids
PPARs: Peroxisome proliferator-activated receptors
OMM: Outer mitochondrial membrane
ACSS2: Acyl-CoA synthetase short-chain family member 2
VDAC1: Voltage-dependent anion channel 1
ACSL1: Acyl-CoA synthetase long-chain family member 1 gene
SOAT1: Sterol O-acyltransferase 1 gene
FMD: Fasting-mimicking diet
RUNX3: Runt-related transcription factor 3 gene
BED: Bromodomain and extraterminal domain
APC/C: Anaphase-promoting complex/cyclosome
EMT: Epithelial–mesenchymal transition
OVOL2: Epithelial transcription factor Ovo-like 2
ETV4: ETS variant transcription factor 4
RNF2: Ring finger protein 2
Sqstm1: Sequestosome-1
RAB1B: Ras-related protein Rab-1B
IGF2BP1: Insulin-like growth factor 2 MRNA-binding protein 1
YAP: Yes-associated protein
FABP: Fatty acid binding protein
SOX2: SRY-Box transcription factor 2
OCT4: Octamer-binding transcription factor 4
ZDHHC4: Zinc finger DHHC-type Palmitoyltransferase 4
PROTAC: Proteolysis-targeting chimeras
FA: Fatty acid
